# Designing and Implementing the MySusCof App—A Mobile App to Support Food Waste Reduction

**DOI:** 10.3390/foods11152222

**Published:** 2022-07-26

**Authors:** Rainer Haas, Hakan Aşan, Onur Doğan, Claus Rainer Michalek, Özlem Karaca Akkan, Zeki Atıl Bulut

**Affiliations:** 1 Department of Economic and Social Sciences, Institute of Marketing & Innovation, University of Natural Resources and Life Sciences, Vienna, Austria; 2 Department of Accounting and Tax Applications, Izmir Vocational School, Dokuz Eylul University, Konak 35220, Turkey; hakan.asan@deu.edu.tr; 3 Department of Management and Organization, Izmir Vocational School, Dokuz Eylul University, Konak 35220, Turkey; onur.dogan@deu.edu.tr; 4 Teaching and Learning Services, University of Natural Resources and Life Sciences, Vienna, Austria; claus-rainer.michalek@boku.ac.at; 5 Department of Electronics and Automation, Izmir Vocational School, Dokuz Eylul University, Konak 35220, Turkey; ozlem.karaca@deu.edu.tr; 6 Department of Marketing and Advertising, Izmir Vocational School, Dokuz Eylul University, Konak 35220, Turkey; atil.bulut@deu.edu.tr

**Keywords:** mobile app, food waste, consumer behavior, food consumption

## Abstract

Consumers are responsible for almost 50 percent of food waste. Consumer-focused interventions are crucial to achieve many Sustainable Development Goals (SDGs), especially SDG 12.3. There are many factors that cause food waste, and these can be prevented by changing the consumption behavior of adults. Mobile apps are seen as promising tools to change consumer behavior for ensuring more sustainable food consumption. This study describes the development process and examines the perceived quality of MySusCof, an app intended to reduce the food waste of consumers. The uMARS scale was used for collecting data from consumers. Within the scope of the study, two studies were conducted to examine the development process of the application and to determine the user reactions to the mobile application. Results show that gamification elements with hedonic and social components, as well as functional aspects, are important features for user engagement and perceived impact. The qualitative results also supported the user experience in both hedonic and functional value and role of mobile apps to lead behavior change. This study serves as a guideline for future developers of mobile apps intended to lead consumers to a more sustainable food consumption.

## 1. Introduction

EU member states have to reduce 50% of its per capita food waste at the retail and consumer level by 2030, and reduce their food losses along the food production and supply chains [1], to reach the target of the Sustainable Development Goal 12.3. It is estimated that over 50% of food waste in Europe originates at a consumer level [2]. Consumer-focused interventions are crucial to achieve the ambitious goals to reduce food waste.

There are several drivers for consumer food waste. For instance, lack of planning around food shopping, insufficient communication between household members, good provider identity, refusing to accept imperfect food [3], over-purchasing [4], poor menu planning [5], oversized packaging [6], lack of cooking skills, etc. Additionally [7], absence of consumer awareness, deficiency of knowledge among stakeholders, and lack of awareness to promote sustainable consumption are significant barriers towards adoption of food waste reduction. Many factors causing food waste could be prevented by changing adults’ consumption behavior. However, the majority of interventions are information campaigns, with little evidence about their impact [8,9]. The EU action plan on Food Waste recommends to “promote and facilitate the use and development of a wider range of behavior change methods, through an active collaboration between researchers and practitioners. The methods should focus on influencing social norms to trigger behavior change, rather than only providing information and increasing awareness on the issue” [1].

The use of technology in the form of smartphone apps is a possible alternative to sole information campaigns and is increasingly acknowledged as a significant mean to support food waste reduction [3]. For example, the app “Too good to go” connects consumers with supermarkets, coffee bars, bakeries, and restaurants who want to give away food for reduced prices instead of throwing it away [10]. The German “Beste Reste-App” (i.e., best food leftovers) provides consumers with recipes to cook leftovers [11]. The “Food Loop App”, financed by the EU commission, connects retailer inventory systems with a consumer app to inform consumers about reduced prices for food products with an impending “best before date” [12]. Other apps promote food sharing by connecting consumers or local businesses [3]. Overall, apps focusing on reducing food waste could be seen as a new tool to nudge consumers towards a more sustainable lifestyle. Kameke and Fischer [13] have shown that nudging consumers to use purchase plans for food shopping has positive effects on food waste reduction. They also identified in their study that consumers are looking for specific advice on meal planning, on feedback on their individual food waste behavior, and on social interaction with their community [13]; all together, features which could be provided over an smartphone app.

New technologies such as mobile apps have the potential to more effectively educate about the complex, social, environmental, and economic challenges that come with a transformation towards a more sustainable society. Practices are useful for combining traditional cognitive (intellectual) learning with physical, emotional, or spiritual learning, and they take into account the specific institutional contexts that characterize societal challenges [14]. In the context of socially responsible or sustainable behavior, Laasch et al. [15] demonstrated how app-enabled learning is able to create real-life actor networks that in turn enact socially responsible behaviors. Similarly, in a recent review on sustainability management teaching resources Aragón-Correa et al. [16] revealed that despite the importance and high level of satisfaction with applications and new digital resources, instructors often only marginally use these mobile applications and resources within the existing array of teaching resources. 

Mobile apps designed for reducing food waste can be classified into two categories: food redistribution and household food management apps [17]. There are various food redistribution apps that aim to redistribute food before it becomes waste, such as apps including sales-to-purchase, peer-to-peer sharing, and providing donation [18]. For reducing food waste, sales-to-purchase apps such as “Too good to go” [10], “MyFoody” [19], and “Leloca” [20] may sell products at discount prices that, for different reasons, are likely to be thrown away, may be about to expire, or have manufacturing defects. Olio, a UK-based food sharing initiative, can be given as an example of peer-to-peer sharing apps [21]. Food banks, which collect food from retailers and distribute it free of charge to those in need, are examples of providing donations.

Household food management apps aim to reduce waste by assisting individuals with inventory control, purchasing, and recipe management rather than sharing or redistributing food. For a potential additive outcome, social media and smart devices can be linked to these mobile app systems.

Mobile apps that focus food redistribution are used to connect groups and individuals to redistribute food owing to the speed, flexibility, and relatively low cost of operation which deal with the perishability and fragility of food [22]. Knowledge of available household consumable food, knowledge of the location in which the available consumable food is contained, and food literacy, the understanding of how to use the available foods, are the primary factors that may contribute to prevent food waste at the household level [23]. Household food management focused apps have three subtypes: alert system, visual stock list, and recipe recommendation [17]. This demonstrates that a well-designed mobile app may raise awareness of food waste for some individuals and households, support them to change waste behaviors, redistribute leftovers, and change cultural and social norms about food waste. For example, the impact of Too Good to Go, the well-known mobile app, on reducing food waste was investigated in [24]. After using the app, respondents stated that they started to take actions to reduce food waste such as buying less groceries, cooking more creatively with leftovers, freezing food or leftovers more often, doing grocery shopping more consciously, and cooking less food by basing amounts more on how much food is actually needed. Thus, it can be expected that the high level of perceived impact of mobile apps may support individuals to change behaviors for more sustainable food consumption.

The aim of this paper is to understand the perceived quality as well as the subjective quality and perceived impact of MySusCoF app, which is developed for raising awareness on sustainable food consumption. Thus, the experiences and findings of this research may serve as guidelines for future app development projects, aiming to promote a better sustainable lifestyle of consumers [25]. The paper is structured in the following way. The theoretical background gives an overview of the potential of app technology to stimulate behavioral change, followed by the materials and methods chapter explaining the mobile app development process and the usability evaluation. In this context, determinants of the quality of the mobile app were also investigated. Two studies were conducted in order to examine (1) the developing process of the app and (2) to determine user responses towards the mobile app. The results section contains the usability evaluation, finishing with the discussion and a future outlook for further research.

## 2. Hedonic-Utilitarian-Hybrid Mobile Apps

The use of technology creates positive impacts in many aspects, such as facilitating communication and socialization in human life, accelerating access to information and reducing information asymmetry, and saving time and cost in many works in daily life. On the other hand, it can have negative effects, such as technology addiction, breach of privacy and protection of personal data, and creating a digital divide. In addition to these and many other impacts, one of the most common impressions of the increase in both the penetration rate and the frequency and duration of the use of technology is the opportunities it offers to consumers in terms of searching for, accessing, and using information. Thanks to technologic developments and adoption, mobile apps have become one of the routine tools of daily life, with two types of beneficial impacts: functional and emotional. 

Mobile apps are classified based on their nature into two categories, named hedonic/experimental and utilitarian/goal-oriented. There are usually three alternative approaches to a mobile app: utilitarian orientation, hedonic orientation, and hybrid. For example, banking and productivity apps support utilitarian/targeted outcomes, while games and social networking apps support hedonic/experiential outcomes. With utilitarian apps consumers mostly seek information, i.e., these applications are task-oriented, rational, and considered work-related, while hedonic mobile apps are used by consumers to pass time in activities such as shopping, entertainment, and playing games [26]. 

Many mobile apps were focused on utilitarian aspects such as functionality and sharing information, especially serving as an online learning tool for a wider audience. Some others were more hedonic oriented and focused mostly on aesthetics and engagement. According to prior research, users’ perception of the values, such as emotional, hedonic, or utilitarian values, influence user satisfaction [27], user adoption and intention to upgrade [28], behavioral engagement with mobile apps [29], and continuance or purchase intention [30,31].

A hybrid approach can also be used for mobile apps by using both hedonic and utilitarian values. Morgan-Thomas and Dudau [32] define participation in online learning as a multidimensional construct that includes behavioral, affective, and cognitive elements. Behavioral engagement is related to learning activities and interactions with learners and instructors. Affective engagement refers to the response to the course or lecturer and higher-order affects such as hope, pride, or feeling confident. Cognitive engagement covers thinking, planning, and self-regulating for one’s learning and perceived understandings, knowledge, and mastery. Kim et al. [33] found that mobile engagement intention was established through motivation, perceived value, and satisfaction, which can be related to affective engagement elements. They also emphasized that user satisfaction is driven by high usability of the app. High usability leads to high user satisfaction, which leads to high engagement intention. Besides, “hedonic and social” motivations were found to be highly related to continued engagement intention. On the other hand, Kim and Baek [34] show that perceived engagement is the strongest predictor of mobile app engagement. It has been determined that consumers interact with mobile applications at a high level when they have mutual and interactive experiences with their preferred applications. Time convenience, interactivity, and compatibility positively increase mobile app engagement. Informational and experiential apps have a positive moderating effect on the afore mentioned time convenience and interactivity. This shows that interaction as a predictor of behavioral engagement has a strong impact on using mobile app. In addition, it is predicted that compatibility increases mobile application interaction and compatibility is not related to the personality characteristics of the end user of mobile applications, but to the situation where original and customized content can be suitable for user needs. In contrast, effort expectancy was found to have no direct effect on mobile app engagement.

In respect to this classification, the MySusCof app is a hybrid app with utilitarian benefits by providing information about how to reduce food waste and with hedonic/experimental outcomes by providing game elements such as quizzes and rewards by collecting bonus points in form of kudos. A gamification method was used to apply hybrid approach while developing the MySusCof app in order to ensure providing both utilitarian aspects and hedonic aspects. 

The field of gamification has been the subject of increased scientific research since about 2010/2011 [35,36]. Huotari et al. focused on the goal rather than the methods when they proposed the following definition: “Gamification refers to a process of enhancing a service with affordances for gameful experiences in order to support users’ overall value creation” [37]. Referring to Ryan et al., they felt it was important to emphasize that pure behavior change without the gamified experience, which is based on voluntariness, would limit users in their freedom of will [38]. In terms of awareness and consumer behavior, there are indications that gamification could be a promising approach, for example also in the area of food waste, however there are some barriers that prevent people from behaving accordingly in reality, for example because of store promotions [39,40]. However, despite the context of supermarket shopping, Xi et al. reported in their experiment that the gamification group had higher self-reported awareness of wasting food and lower food wastage than the control group. Based on their study, they see promising evidence for gamification as an effective education tool [39].

Gamification also opens up new opportunities in the mobile app sector to induce behavioral change. For example, Douglas et al. studied the area of sustainability, and although they were unable to determine why certain mobile apps were more effective than others, they generally saw gamification as a promising method for success [41]. Beck et al. examined 2400 mobile apps from the energy use domain and found 57 that contained gamification elements; however, they concluded that most of these applications had major deficiencies in the design and implementation of these of gamification and game design elements and as well as behavioral constructs; the latter would be very important when it came to sustainable behavior change [42]. Additionally, from the field of sustainable energy use comes a study by Whittaker et al., who used the “Reduce Your Juice” mobile app to demonstrate the extent to which the flow experience, as part of the user experience, increases the value perceived by users to influence their sustainable behavior [43]. Since engagement with the mobile app is important to retain users, attention must be paid to satisfying the basic psychological needs for competence, autonomy, and relatedness to other users [44]. Since the game elements of achievement and progression are particularly effective, Bitrián et al. [44] recommend challenges and real-time feedback in addition to the classic elements points, badges, leaderboards. Based on these finding, the developers of the MySusCof app decided to included elements of achievement and progression, naming them “adventures”, with feedback providing the users a benchmark about their own food waste behavior.

## 3. Methodology

From 2018 to 2021, the Erasmus+ project “SUSCOF” aimed to assess and change adults’ behavior on sustainable consumption of food (including food waste behavior) in four European countries and one non-EU member state (Austria, Denmark, Sweden, Slovakia, and Turkey) (“Assessing and changing adult’s behavior on sustainable consumption of food”) [45]. At the first stage of the project, expert interviews in the partner countries served as a basis to understand the needs and areas of deficiencies in respect to food waste. In the second stage, sustainable food consumption behaviors of adults in the partner countries have been measured by a quantitative survey to calculate a comprehensive index on sustainable consumption of food (SCOFI). In the third stage of the project, the preparation of training materials and the development of the “MySusCof” app was the main output. 

Mobile app developers can easily improve the features and characteristics of their app. A mobile app’s release life-cycle is also often shorter than other products. For this reason, mobile app developers are often more interested in looking for possible improvements of the mobile app [46]. Application stores and platforms that offer applications to users provide an opportunity for users to review applications and share their experiences. Users can provide feedback on a particular app they use or download in the form of quantitative ratings or textual reviews. This type of feedback allows users to share their opinions and experiences about an app that might motivate or deter other users from downloading an app [47]. Apart from user feedback on application stores or platforms, more detailed feedback can be achieved using assessment tools. MARS (Mobile Application Rating Scale) is the well-known and most used scale for evaluating the quality of the mobile app. As it is based on the feedback from professional use of researchers, scientists, or specialists, a user-friendly version of the MARS, uMARS, was developed in order to gain training-free feedback from regular users [48]. uMARS is the app user version of the original MARS for evaluating the quality of the app. The uMARS has 16 items in four dimensions named engagement, functionality, aesthetics, and information [49]. Functionality and information can be related to utilitarian value, while engagement and aesthetics are determinants of hedonic value.

The research was done in three steps. First, a mobile app, MySusCof, was developed in line with the Dynamic Software Development Method (DSDM). Second, we examined a pilot study for the beta version of the app. Third, a usability study using a mix method was done with users in order to understand the consumer response and the perceived quality of the app.

### 3.1. DSDM Process for Developing Mobile App

A common problem during software or app development is that evaluation of the software by its end-users takes place ***after*** the software/app is finished and not during the design process [50]. Making changes at the end of the design process are cost intensive and often suboptimal in respect to the usability of the product. Therefore, the SUSCOF consortium decided to follow an iterative design process by developing a prototype version of the MySusCof app, which was then evaluated with a usability test, and the results were included in an improved version before deployment of the app. This approach follows the concept of DSDM as a guideline for software development. The advantages of the DSDM method are; (a) early customer involvement, (b) iterative development including user feedback, (c) self-organizing teams, (d) adaptation to change [51]. According to [52], there are some principles underlying DSDM, such as user involvement, empowering the project team, frequent delivery, addressing current business needs, iterative and incremental development, allowance for reversing changes, high-level scope being fixed before project starts, usability testing throughout the life-cycle, and efficient and effective communication. The development of an app needs feedback from the specialist as well as end-users. To collect feedback from the end-users, a usability test was applied during the development process.

**Figure 1:**
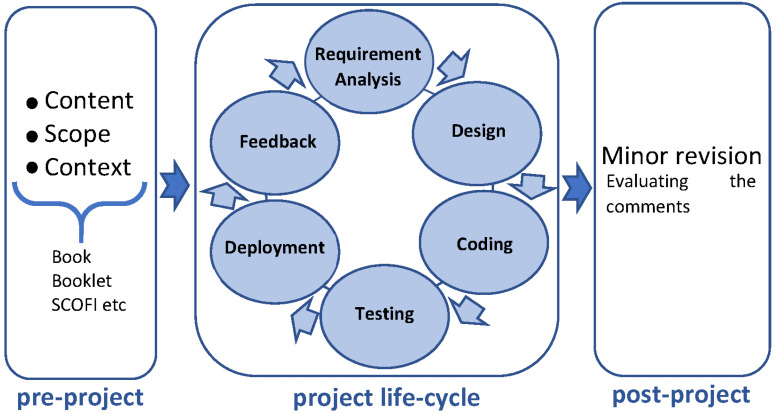
Project Implementation Process.

DSDM divides projects in three phases: pre-project, project life-cycle, and post-project [52]. Figure 1 shows the derived process for developing MySusCof app.

The steps have been executed as follows.

#### 3.1.1. Pre-Project

In a first step, the main structure of the app, the scope, context, and content of it was defined. The scope of the app is to create awareness about the importance to reduce food waste and to change behavior towards a more sustainable food consumption lifestyle. The context of the app is to engage adults in a way which is educating and entertaining about the topic of food waste and sustainability.

For the content we used books, booklets, and videos as educational material in the application, which have been produced by the SUSCOF research consortium. The presented information was combined with entertaining aspects to increase user engagement with the app. For this reason, a content supported by quizzes has been created that shows what people should do in each phase—before consumption, during consumption, and after consumption—to make their food lifestyle more sustainable. This content in the app is called the food consumption adventure and it consists of three main stages (shopping trail, kitchen trail, and leftovers) and various sub-stages.

Finally, gamification-based ideas have been implemented to make all this content more usable. Mobile app users can collect kudos by finishing training modules, reading, or watching the materials in the app, promising different statements about more sustainable food consumption, and doing many other activities in the app. In this gamification sense, the “forest” analogy is applied. There are five different levels in the analogy: seed, sprout, sapling, tree, and forest. Users start their more sustainable food consumption adventure on the seed level and level up by collecting kudos by doing several activities in the mobile app.

#### 3.1.2. Project Life-Cycle

Project life consists of six phases: requirement analysis, designing, coding, testing, deployment, and feedback.

##### Requirement Analysis

Requirement analysis is handled in two parts: content needs and technical needs. First, the content needs have been identified by expert interviews during the SUSCOF project [53]. The findings of the expert interviews during the SUSCOF project have revealed that adults’ food consumption and food waste behavior is not sufficiently sustainable in partner countries, especially in Slovakia and Turkey [45]. However, the experts also emphasized that with concerning food waste behavior of consumers in Austria, Denmark, and Sweden, there is significant potential for improvement. Unplanned shopping and impulse buying, false believes about “best-before” dates, inappropriate storage, lack of knowledge about cooking food, and alternative uses of food are key factors behind food waste, according to the experts [45]. Based on these findings, a book, booklets, videos, and training materials have been developed during the SUSCOF project and served as basis for the content creation of the app.

Second, technical requirements have been exhibited. While developing the app, Microsoft Visual Studio 2019 as application development platform and relational database management system MsSQL 2018 Expression as databases were needed. In addition, different operation systems which run on different devices (tablets and mobile phones) were needed to test the application. During the application development phase, mobile view tests of different browsers were made in order to understand how it looks on the web as well. Finally, it was decided to handle the content in accordance with the gamification idea.

##### Designing

It is the aim that the MySusCof application works flawlessly on all devices. For this reason, the application is designed in a responsive structure. Computer, mobile phones (platform independent), and tablets can use the application with appropriate resolution and functionality. Small adaptations are made on this structure for application markets.

The application is designed in a modular structure. Each function of the application (SCOFI, Take Action, Find Out More, How am I Doing?) is designed as different modules and communicates with the main application. Services are used for communication. In this way, it can develop and update separately for each module.

The entity framework was used as the database application model. In this way, the application can be integrated with different databases if desired. Currently, MsSQL database, which is a relational database management system, was used.

**Figure 2:**
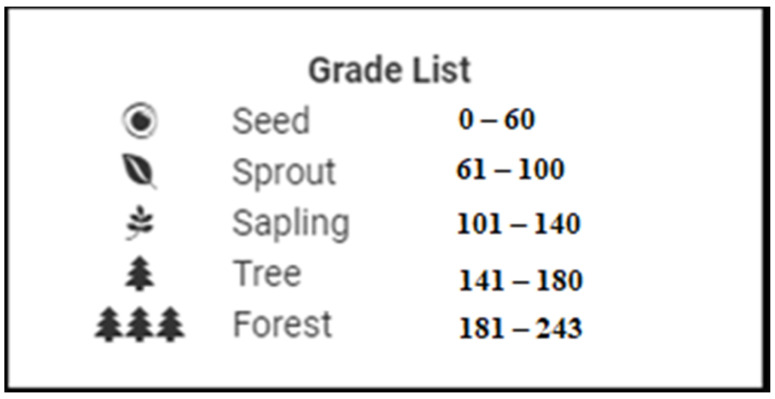
Kudos Grade List.

Users can earn kudos by attending classes, making promises, schooling documents, taking quizzes, and watching videos. The progress levels of the users are given in Figure 2.

**Figure 3:**
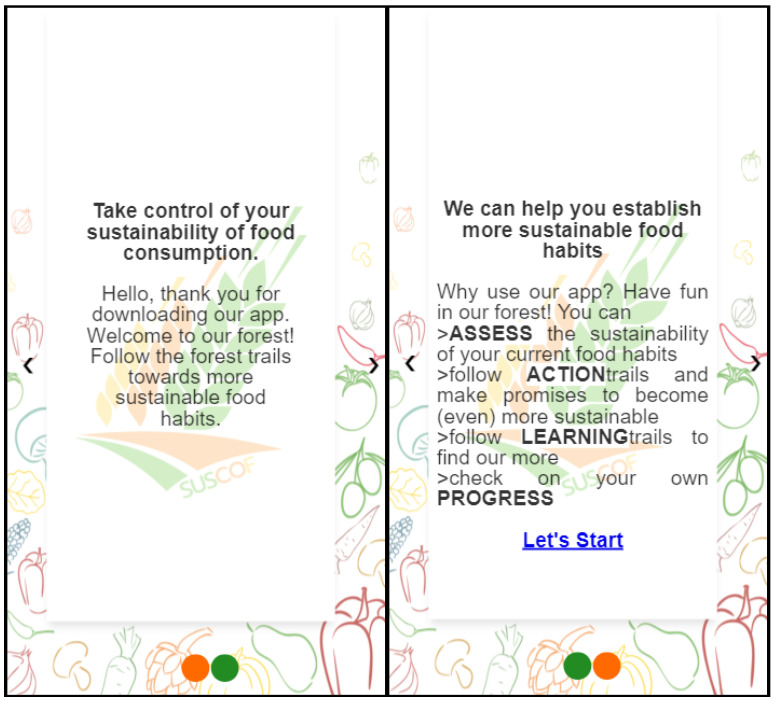
Information Page.

The application opens with an information page during its first use (Figure 3).

**Figure 4:**
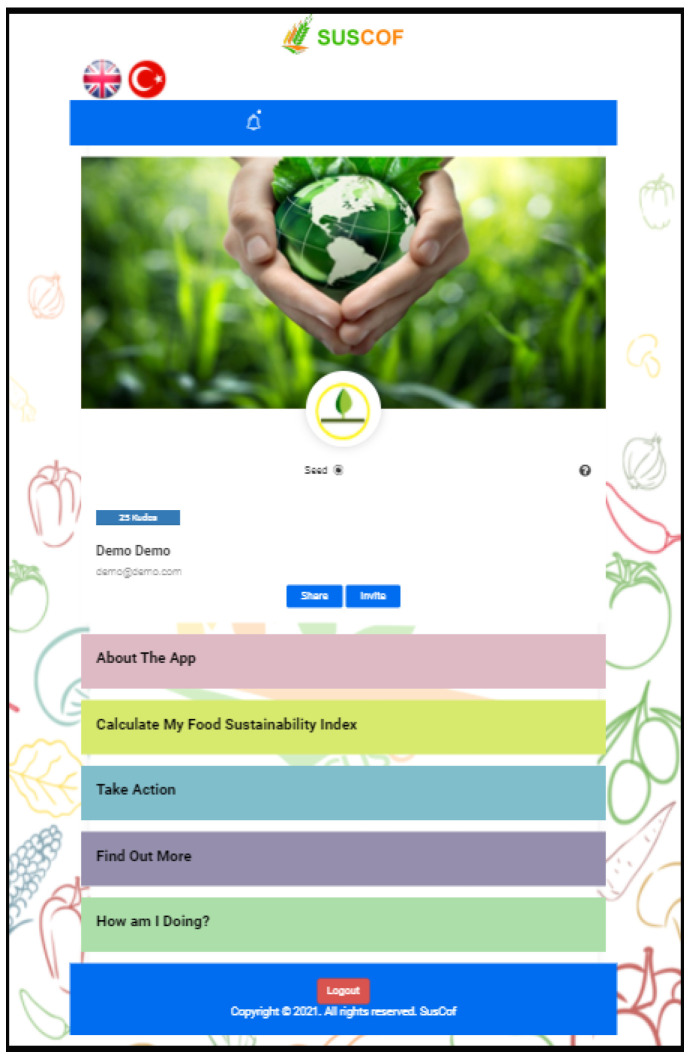
App Home Screen.

The aim of the information (landing) page is to inform the user about the program. The app home screen consists of five main menus, which can be seen in Figure 4.

**Figure 5:**
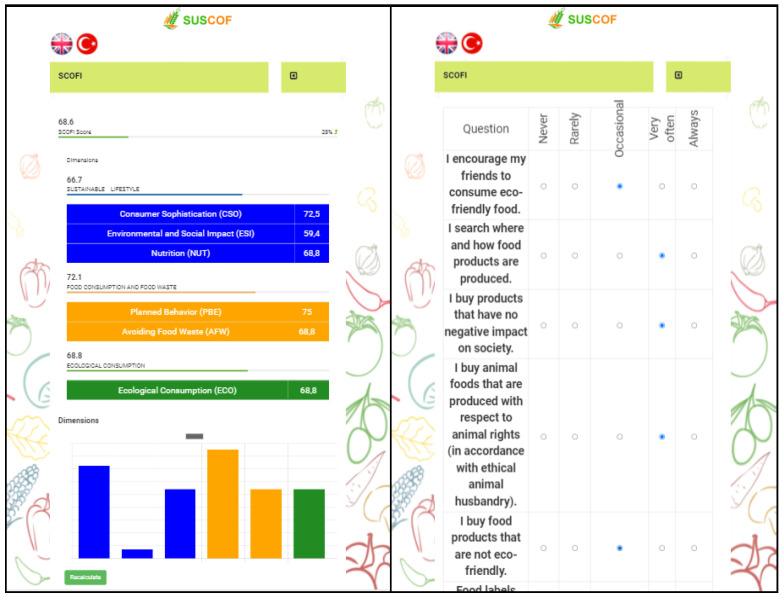
SCOFI Display.

Users can also see their progress in the progress bar. “About The Application” section gives detailed information about the mobile app. “Calculate My Food Sustainability Index” section calculates the user’s SCOFI score. In addition, users are informed with visual elements (Figure 5).

**Figure 6:**
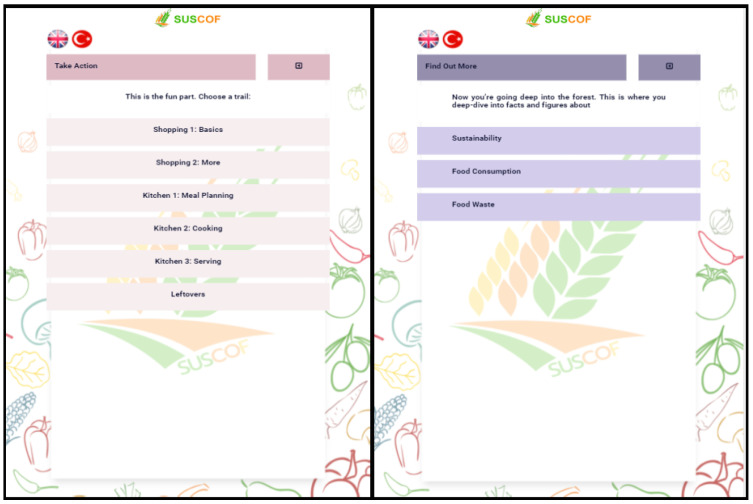
“Take Action” and “Find Out More” Screen.

As can be seen in Figure 6, users can attend training modules named “take action” and “find out more”.

**Figure 7:**
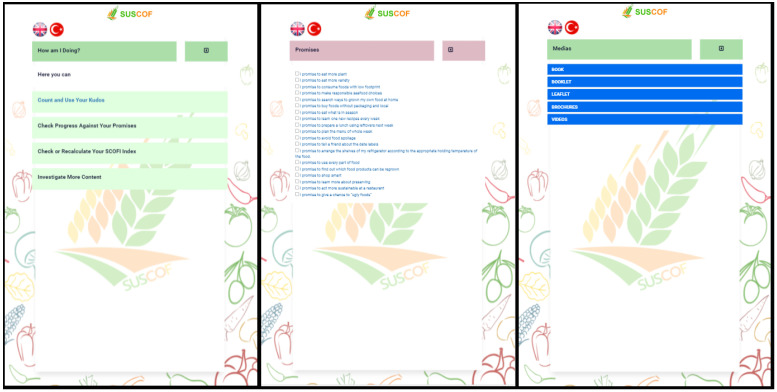
“Investigate More Content” Screen and Contents.

“How am I Doing?” section includes “Count and Use Your Kudos”, “Promises” section, and “Investigate More Content” section (Figure 7). Kudos earned in the “Count and Use Your Kudos” section are displayed in chronological order. In the “Promises” section, users can make promises about the sustainable consumption of food. In the “Investigate More Content” section, users can access various documents and videos.

##### Coding

HTML 5 language was used for the front-end coding of the application. JavaScript language was used to support this language. The Boostrap front-end library was used to run the application on different platforms (https://getbootstrap.com/ accessed on 30 May 2022). Net language was used in the back-end programming of the application. The Net language is an OOP-object-oriented programming language developed by Microsoft from the C language. The application is coded using the Visual Studio 9 platform. Coding tests were conducted with various browsers and emulators.

The application was developed in Net language, on the Visual Studio 2018 IDE. MsSQL 2018 was used as database. jQuery language is used for client side processes. A connection is made between the software and the database with the entity framework. This method provides flexibility in database changes in the future. Since the application will be viewed on different platforms, the bootstrap library has been used in order to have a responsive visuality.

##### Testing

The MySusCof prototype was evaluated with a usability test with end-users and the results of the usability test were used to improve specific features of the app. Bevan and MacLeod [54] define usability as quality of use in a specific context as “…we should think of usability in terms of the quality of use of an interactive system by its (intended) users for achieving specific work goals and tasks in particular work environments”.

**Figure 8:**
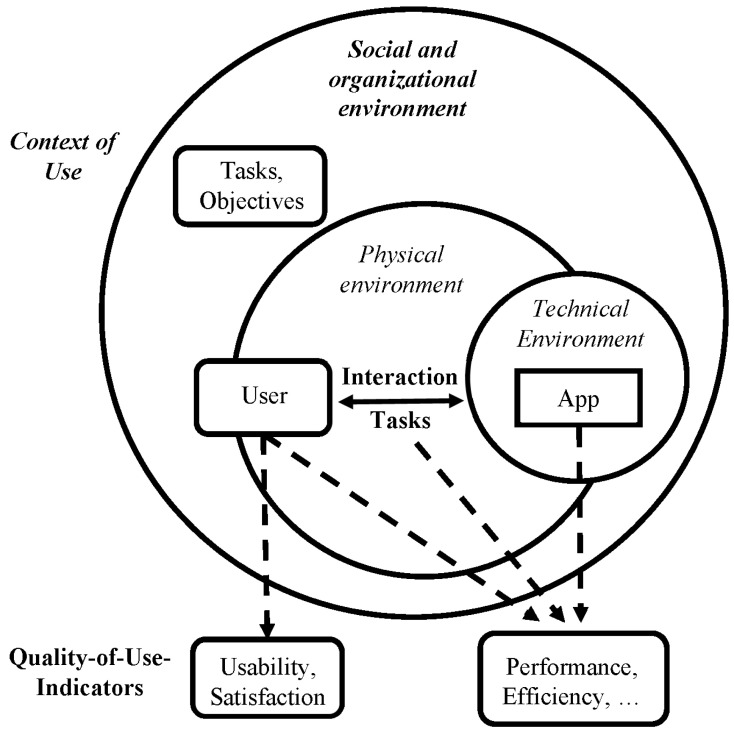
Usability as Quality of Use in a Context (Source: [54,55]; own depiction).

The quality of use model assumes (see Figure 8), similar to the task-technology-fit model of Goodhue [55], that the usability is a result of interactions between the user, the physical and social environment, the technical infrastructure, and the tasks. The usability of an app is not only influenced by the app itself but also by characteristics of the user, the posed tasks, the technical infrastructure (operating system, processor power, quality of internet connection, …), and the physical environment in which the app is used (light conditions, temperature, background noise, …).

A major advantage of qualitative usability methods is that small sample sizes are sufficient to identify the majority of usability errors. The optimal sample size is dependent on the homogeneity of the user group and the demands of the software developers on the percentage of identified errors [56,57,58]. Virzi [58] showed in an experimental setting that the number of identified errors with each additional user follows a curve of diminishing returns. He demonstrated that 80% of usability errors can be identified with four users. The probability to identify new usability errors with an additional user is diminishing. The most severe usability errors are identified with the first users.

Bevan and Macleod [54] recommend, for pragmatic reasons, to include up to ten users. Testing of groups of up to ten users is generally found to be a practical proposition, as the cost and time for testing larger groups is often prohibitive. With five users it is possible to identify 84% of usability errors, and with ten users it is possible to identify 98% of usability errors [58].

### 3.2. Study 1: The Pilot Study

The objective of the pilot study was to identify minor to severe usability errors and to get information about the overall appeal of the app. Furthermore, the aim was to see if the users find the content helpful to improve their food waste and sustainable consumption of food behavior.

The pilot study recorded the probands during a beta-test via video and asked them for feedback afterwards. A beta-test evaluated software with a selected group of users in an everyday life setting, as opposed to a more artificial setting in a usability laboratory. After the test, the probands filled out uMars, a standardized scale from Stoyanov et al. [49]. uMARS scale has three sections: app quality ratings, app subjective quality, and perceived impact. App quality ratings contain four subscales measuring engagement, aesthetics, functionality, and information quality. The **engagement** dimension subscale contains five questions, including entertainment, interest, customization, interactivity, and target group. **Functionality** dimension includes performance, ease of use, navigation, and gestural design. **Aesthetics** dimension includes layout, graphics, and visual appeal, while **information** covers quality of information, quantity of information, visual information, and credibility of source. App subjective quality was measured by using four items about recommending the app, intention to use the app in the future, intention to pay for the app, and overall (star) rating of the app. The uMars scale includes five questions about the perceived impact of the app to change behavior.

The probands were allowed to use their own smartphones. Before the pilot study, the users were trained about the uMARS scale and mobile app development process. Each test person had to go through a set of tasks such as registering, filling self-assessment test, completing learning adventures, etc., while being accompanied by two additional researchers, one taking notes and one recording the test via video. The probands were told to think aloud while using the app and were encouraged to comment on their experience while using the app. After the pilot study each user was asked about their general impression with the app, and if there were additional critique or proposals of improvement. At the end, each user filled out a survey based on the user version of the mobile application (uMARS) rating scale from Stoyanov et al. [49,59].

### 3.3. Study 2: The Usability Test

After the pilot study, the usability test was done with consumers. uMARS scale was used to collect data from consumers using Google Forms. An online form was prepared and distributed using non-probability convenience sampling method. An instruction guide was first provided to the users that explain how to download and use the app. Some assignments were defined for users in order to be ensured that they experienced almost all menus and content. Afterwards, users answered 5 point Likert type questions in app quality ratings in four dimensions named engagement, functionality, aesthetics, and information, as well as subjective quality of the app, perceived impact, and demographic questions.

## 4. Results

The results consist of two parts: first, we describe the pilot study results, and second, the usability test results.

### 4.1. Results of Study 1: Pilot Study

The MySusCoF app was tested with 11 users in October 2021 at the University of Natural Resources and Life Sciences in Vienna. With 11 test people, it is possible to detect 98% of all usability errors [58]. Each project partner of the SUSCOF project was responsible to recruit test persons. Therefore, the probands were from different nationalities, stemming from Austria, Denmark, India, Kosovo, Slovakia, Sweden, Germany, Italy, and Turkey. The international composition of the test persons is in accordance with the intended global user group of the app. Seven of the probands used an Android smartphone, four used an iPhone. Table 1 shows the demographic profile of participants.

In the sample, there were four participants between 18 to 30 years old, five between 31 to 50 years, and two over 51 years. There were more females than men in the sample, and the education level was—due to the fact that most of the probands were affiliated with universities—higher compared to the education levels in the EU.

The results of the study 1 are structured in the following way. First the results of the uMars are explained, then examples of user feedback after the test are given in detail.

#### 4.1.1. Results of the App Quality

**Figure 9:**
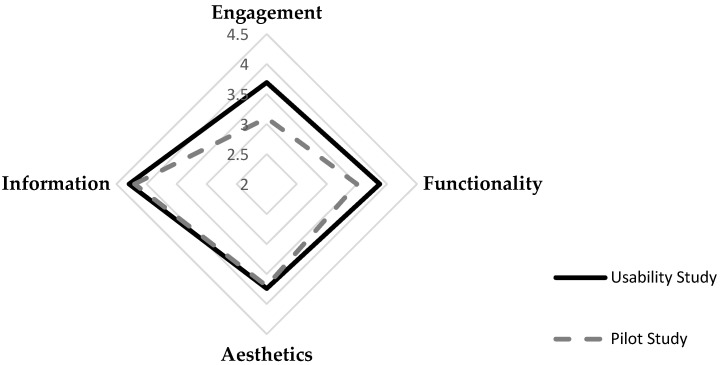
Mean Scores of Engagement, Functionality, Aesthetics, and Information in Two Studies.

Figure 9 shows the arithmetic mean values of the four objective quality subscales of the uMars: Engagement, functionality, aesthetics, and information. The quality subscales are measured with a scale from one to five, five being the best rating. Each subscale consists of a subset of three to five questions. The overall app quality score is 3.62, representing middle-high level.

The average engagement mean score over these five questions is 3.13. The corresponding item statements related to these ratings are for entertainment “Okay, fun enough to entertain user for a brief time”; for interest “Moderately interesting”, for customization “App allows little customization and that limits app’s functions”; for interactivity “App contains basic interactive features”; and concerning the target group it is “designed for the target group, with minor issues”.

The average functionality mean score over these four questions is 3.50. The corresponding item statements related to these ratings are for performance “App works overall. Some technical problems need fixing, or is slow at times”. For ease of use, navigation and gestural design the average answers were “easy to learn”, “easy to understand”, and “mostly consistent/intuitive with negligible problems”.

The average aesthetics mean score over these three questions is 3.67. The corresponding item statements related to these ratings are for layout “Mostly clear, able to select/locate/see/read items”; for graphics “High quality/resolution graphics and visual design”; for visual appeal “pleasant–seamless graphics”.

From all four subscales, the information subscale got the best ratings. The average information mean score over these five questions is 4.20. All four sub-questions are rated above 4, with credibility of the source the highest (4.36) and visual information the lowest (4.0) from this subset of questions.

#### 4.1.2. Results of the App Subjective Quality

The subjective quality of the app is measured with four questions, all of them ranging from one to five. The first one is “Would you recommend this app to people who might benefit from it?”. Eight of the 11 users would recommend this app to many people. The second question concerning subjective quality is “How many times do you think you would use this app in the next 12 months, if it was relevant to you?”. Nine from 11 test persons said they would use it 3–10 times, and two said they would use it 10 to 50 times in the next 12 months. Asked if they would pay for the app, five probands marked “definitely not”, two answered “rarely”, and four with “maybe”. For the overall rating of the app, six test people rated the MySUSCOF better than average, four as average, and only one as below average.

#### 4.1.3. Results of the Perceived Impact

Ten of eleven probands agreed or strongly agreed that the app had increased their intention to change their food waste behavior and their knowledge about food waste. Eight of ten agreed or strongly agreed that it had improved their attitude and awareness about the food waste topic. When asked if the use of this app will make their food waste behavior more sustainable, the answers are more ambivalent. Five probands said “neither nor” and five agreed or strongly agreed.

#### 4.1.4. Results of User Feedbacks

The recorded videos and the user feedback after the test contained technical errors and comments about the future food waste behavior of the probands. The technical error comments ranged from minor usability errors “font size too small to read”, to severe usability errors, such as some users with an iPhone having reported to be unable to open the links leading to the book or the videos in the app.

“Gaining points for fulfilling tasks is fun, but it is not clear from the beginning what the points are about and for what you get them. The text in the adventures was really small and hard to read. Swiping would be nicer that clicking on the arrow and it wasn’t possible to move back to previous slides. The SCOFI Score needs more explanation, maybe the score can be shown on the home or newsfeed, but it is not clear what to do with it after finishing the survey. I didn’t find the explanations to the promises, only after I was told, I understood I can click on them. I think it is a fun app, that I would use in buses trains and click through for a few minutes”. Another user mentioned “Had Problems to find SCOFI Scores of Sub dimensions. Wrong Wording “food couples” instead of “food categories”. The text is too formal and abstract. More specific advice would be better. Liked the possibility to collect kudos. Would be nice to see how other users are doing with their food waste behavior”.

Concerning future food waste behavior, exemplary comments were “I’m going to cook smaller portions to save food from going bad”, “I plan to improve my planning for meal cooking and will use shopping lists more often”, “I will pay more attention to reducing my food waste concerning bread, fruits, and vegetables”, “I will do more information search about the way food is produced”, “I will use less processed food”, “I will try to reuse more of my leftovers”.

The collected data and user feedback will be implemented in a consecutive phase to improve the app. After implementation, a second usability test will be done to see if the changes have led to an overall improvement of the usability.

### 4.2. Results of Study 2: Usability Study

Overall, 121 respondents participated in the usability study, with 53.7% of the sample being male, 57% of them living in a city center, and 61.9% of them students. The distribution of the age ranges from 18 to 52 with the average age of 26,4. Additionally, 44.6% of the respondent had at least short-cycle university degree. A comparison of results of each dimension in pilot study and usability study is presented in Table 2.

#### 4.2.1. Results of the App Quality

According to the results, the average score of the app quality is 3.90, representing almost the high level of perceived quality. Information has the highest score in all sub-dimensions with an average of 4.29. The functionality score is 3.88, followed by aesthetics with 3.74 and engagement with 3.69, respectively. All these scores are higher than the scores in the pilot study, indicating that the final version of the app has higher levels of perceived quality thanks to the implementation of recommendations received from the pilot study. Figure 9 shows the arithmetic scores of the app quality ratings in four sub-dimensions for both pilot study (Study 1) and usability study (Study 2).

Utilitarian value of the mobile app, which was measured by the combination of functionality and information scores, is 4.09, while the hedonic value of the mobile app, which was the combination of engagement and aesthetics scores, is 3.72. These results indicate that the mobile app was perceived more utilitarian than hedonic. However, as two of the scores are relatively high and close to each other, the app could be considering as hybrid as it was expected.

#### 4.2.2. Results of the App Subjective Quality

Subjective quality scores of the app were measured with four questions. The average scores for recommending the app to other people is 3.79, which represents a high level of intention to recommend the app. On the other hand, participants show a low level of willingness to pay for the app, with an average score of 2.17. For intention to use in the next 12 months, 82% of the participants stated that they will use the app at least 3 times in the following 12 months, while 45% of them thought they would use it at least 10 times. The overall (star) rating of the app is 3.71, representing a middle level of satisfaction.

#### 4.2.3. Results of the Perceived Impact

The overall score of the perceived impact of the app is 4.06, representing a high level of the impact on the users to raise awareness, knowledge, and intention to change behavior, in addition to attitude. For each aspect, it was found that the average score for awareness is 4.13; knowledge is 4.14; attitudes is 3.93; intention to change is 4.07; and behavior change is 4.02. This shows that the app has a positive perceived impact on users in all aspects.

#### 4.2.4. Results of User Feedbacks

Further comments about the app from the usability study group show that respondents found that the app is easy to use in general. However, some of the respondents suggested further improvement, such as “Pages have forward button but no back button, which could be added”, “Graphics could be improved”, “Page transitions could be faster”, “Graphics could be clearer”. Besides, some participants stated that “The use of the application is simple, understandable, and fast, and the information transfer is conveyed to the reader at a very good level”, “The app raises awareness, so I like it”. Accordingly, these qualitative findings support the results about the app’s usability.

Concerning future food waste behavior exemplary comments from usability study were “I will pay attention to wastage of water and waste of bread”, “I will use less packaged products”, “I’ll be more careful about waste with the new information I’ve learned from the app”, “Seasonal foods should be consumed so that waste is prevented. Because food is healthier and more beautiful in season”. This shows that the app has an impact on consumers’ intention to change behavior for more sustainable food consumption. This is also consistent with the high level of perceived impact that was found in the usability study 2. Consequently, user feedback about the application is positive, and users stated that they have gained awareness about food waste thanks to the application.

## 5. Discussion

Sustainable consumption of food has become more crucial in recent years due to the rising understanding of long-term impacts of climate change. Many of the UN’s SDGs are related to food consumption because this phenomenon has a large-scale impact on carbon emission, poverty, zero hunger, and economic growth. Thus, keeping in touch with consumers, who are responsible for food waste at a household level, is one of the priorities for achieving more sustainable consumption of food, reducing food waste, and enabling a circular economy. Therefore, based on data collected from 132 consumers in pilot and usability studies, this study focuses on mobile app development process and the impact and perceived quality of the app. Although the app has been developed mostly based on utilitarian outcomes, such as information and functionality, gamification aspects were also adopted for gaining hedonic outcomes such as engagement and aesthetics to attract interest of consumers.

Findings of this study show that the perceived app quality was found high thanks to high scores of information and functionality, which are indicators of a utilitarian value. At the same time, hedonic values of the app were found to be perceived as high, indicating that MySusCof is a hybrid app with both utilitarian and hedonic values. A high level of perceived app quality overall is consistent with the evidence of [33,37], which asserted that users’ overall value creation is supported by gameful experiences, and user satisfaction is driven by high levels of usability. On the other hand, utilitarian aspects of the app are mostly related to cognitive aspects which aim to raise awareness and reduce food waste at home. The high scores in dimensions of utilitarian aspects of the app are consistent with the findings of [14,15,16]. Besides, affective aspects, which are mostly related to hedonic values of the app, were also perceived as high in our study. This is consistent with [39], which provided evidence for gamification as an effective education tool.

The high perceived impact values of the app indicate its potential to raise awareness, knowledge, and the intention to change behavior. This finding is in line with previous research that revealed promising evidence about the impact of gamification in mobile apps to support behavior change [38] and reduction of food waste [3,24,39,40].

The EU action plan on Food Waste recommends “an active collaboration between researchers and practitioners” to develop “a wider range of behavior change methods …” [1]. MySusCof app was developed based on a collaboration between researchers and software developers, which is consistent with the suggestions of [1]. The findings of this research take the findings of [38] a step further by revealing the fact that collaboration between developers and researchers triggers more useful mobile apps in the context of sustainable food consumption.

Usability is an important antecedent for user satisfaction, and leads to high engagement rates [33,37]. The usability test revealed minor and severe usability errors, especially the missing compatibility with iPhone users, who had problems in opening the links leading to in-depth information in form of the book and videos. The findings of qualitative analysis supported this idea in line with the findings of [34], which that highlighted the importance of compatibility and interactivity for mobile app engagement. It is found in this study that engagement is one of critical determinants of app quality. There is still potential to improve user engagement with the existing version of the MySusCof app. The overall ratings for the engagement dimensions were slightly above average, stating that the app is fun, but entertains the user only for a brief time, it is moderately interesting, and allows little customization. The interactivity features are on a basic level, which in sum shows the necessity to improve the app. The need for improvement in user engagement for more better app quality confirms the findings of [44].

Hedonic and social motivations are highly related to continuous engagement with an app [33,36], which underlines the need to improve the social and the hedonic, gamification aspect of the app. The rating for interactivity also underlines that user engagement could be improved by including more social, community-based features, such as improved communication features and better possibilities to connect users interested in reducing their food waste, by offering them features to build their own virtual food waste reduction community. Laasch et al. [15] showed that social network features of apps lead to real-life actor networks, which in turn stimulate socially responsible behavior, such as food waste reduction. The implemented reward features of the MySusCof app in form of kudos are useful gamification features, which were appreciated by the test people in our study. App developers should include reward features, because they have a strong positive effect on user satisfaction based on the study of Xi et al. [36].

The applied methodology in app development and usability evaluation method delivered important insights on how to improve the MySusCof app in respect to engagement, functionality, aesthetics, and information. This supports the [59,60]—that the uMars scale from Stoyanov et al. is a useful and cost effective tool to evaluate mobile apps and delivers specific insights about which areas need to be improved from a user perspective.

According to the findings of this study, 73 percent of users indicated that they have gained awareness about food waste by using the MySusCof app. The app offers the users informative materials such as methods to reduce food waste, recipes to evaluate leftovers, guidelines for smart shopping, and simple tips for kitchen use. The implemented app provides benefits as an informal learning tool that everyone can easily access, free of charge. Thus, it supports lifelong learning and raises awareness of food waste. On the other hand, assessing the intention and actual behavior on sustainable food consumption uncovered the impact of the app on their food waste behavior. All probands agreed that the app had increased their intention to change their food waste behavior in study 1, and when asked more specifically if the use of this app will make their food waste behavior more sustainable, only half of the probands agreed. Similarly, the intention to change score was found to be slightly higher than the behavior change. This could be seen as more evidence for the prevalent intention–behavior gap [61]. However, even if only half of all future users of the app would change their food waste behavior, it could be considered a tremendous success.

Gamification has the potential to lead to behavior change [41] and reduce food wastage of consumers [29], and could be seen as a digital tool of nudging consumers into a more sustainable food waste behavior. Kameke and Fischer [13] describe the positive effect on food waste reduction by nudging consumers to use shopping lists. The MySusCof app informs consumers about a variety of specific practical measures, from shopping lists to the difference of best-before and use-by dates. Additionally, the high level of scores in hedonic value and perceived impact based on gamification elements support the findings of [13,29,41].

## 6. Conclusions

It was found in this research that both utilitarian and hedonic values of the developed mobile app associated with sustainable consumption of food are at high levels in respect to the overall perceived app quality, but utilitarian aspects, information, and functionality were perceived higher than hedonic aspects. This shows that utilitarian values are primarily to ensure perceived app quality. On the other hand, it was found that a hybrid app with both utilitarian and hedonic aspects can achieve high levels of intention to change behavior. The implemented gamification elements are an important part to improve the hedonic value of the app. These results support the idea that a hybrid mobile app containing gamification elements might be one of the most effective combinations to provide usability of a mobile app and user engagement and encourage consumer to intend and change consumer behavior. These results indicate that a hybrid mobile app—containing utilitarian and gamification elements as well—might be one of the most effective combinations to encourage consumers to change their behavior towards more sustainable food consumption.

A limitation of the study is that it is based on a small qualitative and quantitative research. Nevertheless, it provides insights that are useful for other app developers, who should follow the same procedure to achieve a solid basis of information provided to consumers. Further, we recommend collaborations between researchers and software developer to use a guideline for software development similar to the DSDM method the SUSCOF consortium applied. To incorporate user feedback at the early stages of having a prototype available provides important information about flaws and severe software errors, which then could be “repaired” before the public release of the app. Any future sustainable app project should include the possibility of users communicating and connecting with each other to share their experiences or to benchmark their own food waste behavior with others.

Future research should investigate the share of users effectively changing their food waste behavior due to mobile app usage. This could be done either in the form of experimental studies by (i) testing the influence of app usage on the amount of food wastage or by (ii) benchmarking the effect of app usage against conventional information campaigns. Quantitative studies following app users over a period of time while they report their food waste behavior are another option. Finally, future research could investigate which consumer groups with differing demographics, such as age, education, or income, mobile food waste apps, are the best mean to promote a more sustainable lifestyle. The findings of this study can also serve as preliminary insights for researchers and practitioners who are planning to develop consumer-oriented apps to promote a sustainable consumption of food products.

## Figures and Tables

**Table 1:** Sociodemographic variables—sample structure.

		Sample	
		n	%
Age	18–30	4	36%
	31–50	5	46%
	51 or older	2	18%
Gender	Male	4	36%
	Female	7	64%
Education	Middle/Vocational School	1	9%
	High School	1	9%
	Bachelor Level	3	27%
	Master/Doctorate Level	6	54%
Place of Living	Rural	2	18%
	City	8	73%
	City Outskirts	1	9%
Profession	Student	2	18%
	Employee	8	73%
	Entrepreneur	1	9%
Nationality	Danish	1	9%
	Austrian	3	28%
	German	1	9%
	Italian	1	9%
	Indian	1	9%
	Slovak	1	9%
	Swedish	1	9%
	Turkish	1	9%
	Kosovan	1	9%

**Table 2:** Comparison of Pilot Study and Usability Study Results.

Variable	Pilot Study		Usability Study		*p*
	Mean	SD	Mean	SD	
App quality	3.62	0.26	3.90	0.58	0.000
Information	4.20	0.38	4.29	0.47	0.092
Functionality	3.50	0.32	3.88	0.77	0.000
Engagement	3.13	0.37	3.69	0.77	0.000
Aesthetics	3.67	0.37	3.74	0.75	0.333
Subjective quality	3.16	0.50	3.26	0.84	0.280
Perceived impact	4.11	0.43	4.06	0.83	0.412
Hedonic Value	3.39	0.29	3.71	0.70	0.000
Utilitarian Value	3.85	0.33	4.09	0.54	0.000

SD: standard deviation; *p*: statistically significance for *t*-tests.

## Data Availability

Data is contained within the article.
